# Volume 34 Index

**Published:** 2012

## Author Index

Adinoff, B.

Clinical Laboratory Stressors Used to Study Alcohol–Stress Relationships
Vol. 34, No. 4, Pages 459–467

Agrawal, A.

Identifying Genetic Variation for Alcohol Dependence
Vol. 34, No. 3, Pages 274–281

Alim, T.N.

Resilience to Meet the Challenge of Addiction: Psychobiology and Clinical Considerations
Vol. 34, No. 4, Pages 506–515

Ames, G.M.

Prevention in the Military: Early Results of an Environmental Strategy
Vol. 34, No. 2, Pages 180–182
Prevention Interventions of Alcohol Problems in the Workplace: A Review and Guiding Framework
Vol. 34, No. 2, Pages 175–187

Anthenelli, R.

Overview: Stress and Alcohol Use Disorders Revisited
Vol. 34, No. 4, Pages 386–390

Back, S.E.

Childhood Trauma, Posttraumatic Stress Disorder, and Alcohol Dependence
Vol. 34, No. 4, Pages 408–413

Bacon, A.K.

Clinical Laboratory Stressors Used to Study Alcohol–Stress Relationships
Vol. 34, No. 4, Pages 459–467

Bailey, B.A.

Prenatal Alcohol Exposure and Miscarriage, Stillbirth, Preterm Delivery, and Sudden Infant Death Syndrome
Vol. 34, No. 1, Pages 86–91

Bailey, C.R.

Resilience to Meet the Challenge of Addiction: Psychobiology and Clinical Considerations
Vol. 34, No. 4, Pages 506–515

Bakhireva, L.N.

Focus On: Biomarkers of Fetal Alcohol Exposure and Fetal Alcohol Effects
Vol. 34, No. 1, Pages 56–63

Barkley-Levenson, A.M.

Bridging Animal and Human Models: Translating From (and to) Animal Genetics
Vol. 34, No. 3, Pages 325–335

**Figure:**
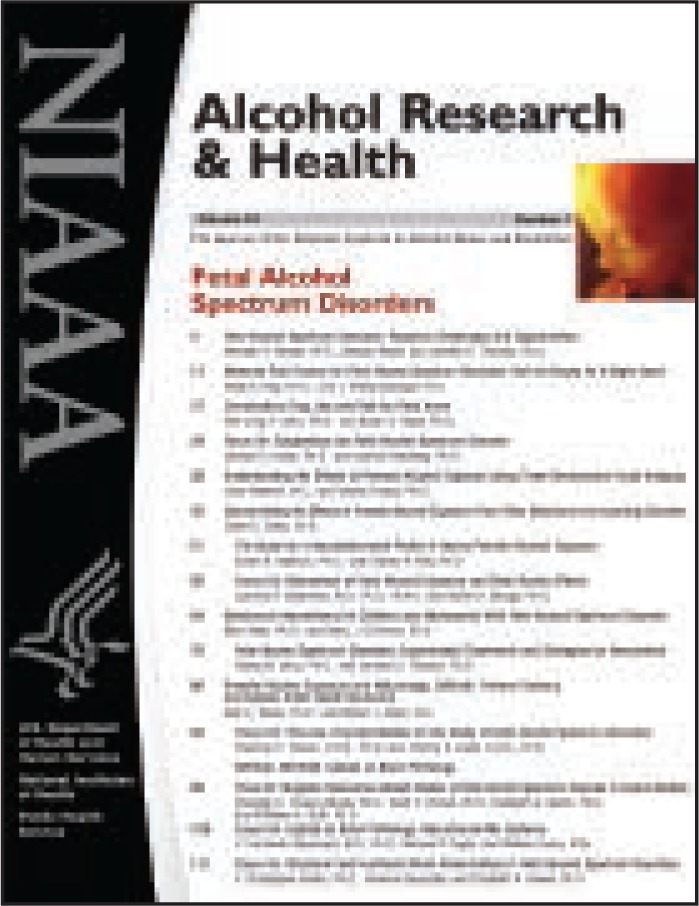
Fetal Alcohol Spectrum Disorders

Becker, H.C.

Effects of Alcohol Dependence and Withdrawal on Stress Responsiveness and Alcohol Consumption
Vol. 34, No. 4, Pages 449–458

Bennett, J.B.

Prevention Interventions of Alcohol Problems in the Workplace: A Review and Guiding Framework
Vol. 34, No. 2, Pages 175–187

Bierut, L.J.

Identifying Genetic Variation for Alcohol Dependence
Vol. 34, No. 3, Pages 274–281

Bloss, G.

The Alcohol Policy Information System (APIS) and Policy Research At NIAAA
Vol. 34, No. 2, Pages 246–247

Borghese, C.M.

Alcohol Dependence and Genes Encoding α2 and γ1 GABAA Receptor Subunits: Insights From Humans and Mice
Vol. 34, No. 3, Pages 345–354

Brady, K.T.

Childhood Trauma, Posttraumatic Stress Disorder, and Alcohol Dependence
Vol. 34, No. 4, Pages 408–413

Buck, K.J.

Discovering Genes Involved in Alcohol Dependence and Other Alcohol Responses: Role of Animal Models
Vol. 34, No. 3, Pages 367–374

Catalano, R.F.

Engaging Communities to Prevent Underage Drinking
Vol. 34, No. 2, Pages 167–174

Chaloupka, F.J.

The Effects of Prices on Alcohol Use and Its Consequences
Vol. 34, No. 2, Pages 236–245

Chard, K.M.

Alcohol and Stress in the Military
Vol. 34, No. 4, Pages 401–407

Chen, W.-J.A.

Combination Drug Use and Risk for Fetal Harm
Vol. 34, No. 1, Pages 27–28

Clarke, T.-K.

Genetic and Environmental Determinants of Stress Responding
Vol. 34, No. 4, Pages 484–494

Coles, C.D.

Discriminating the Effects of Prenatal Alcohol Exposure From Other Behavioral and Learning Disorders
Vol. 34, No. 1, Pages 42–50

Crabbe, J.C.

Bridging Animal and Human Models: Translating From (and to) Animal Genetics
Vol. 34, No. 3, Pages 325–335

Crews, F.T.

Immune Function Genes, Genetics, and the Neurobiology of Addiction
Vol. 34, No. 3, Pages 355–361

Cronce, J.M.

Individual-Focused Approaches to the Prevention of College Student Drinking
Vol. 34, No. 2, Pages 210–221

Cudd, T.A.

Focus On: The Use of Animal Models for the Study of Fetal Alcohol Spectrum Disorders
Vol. 34, No. 1, Pages 92–98

Dawson, D.A.

Defining Risk Drinking
Vol. 34, No. 2, Pages 144–156

Denmark, D.L.

Discovering Genes Involved in Alcohol Dependence and Other Alcohol Responses: Role of Animal Models
Vol. 34, No. 3, Pages 367–374

Dick, D.M.

The Impact of Gene–Environment Interaction on Alcohol Use Disorders
Vol. 34, No. 3, Pages 318–324

Edenberg, H.J.

Genes Contributing to the Development of Alcoholism: An Overview
Vol. 34, No. 3, Pages 336–338
Genes Encoding Enzymes Involved in Ethanol Metabolism
Vol. 34, No. 3, Pages 339–344

Fagan, A.A.

Engaging Communities to Prevent Underage Drinking
Vol. 34, No. 2, Pages 167–174

Feder, A.

Resilience to Meet the Challenge of Addiction: Psychobiology and Clinical Considerations
Vol. 34, No. 4, Pages 506–515

Fell, J.C.

Preventing Impaired Driving: Opportunities and Problems
Vol. 34, No. 2, Pages 225–235

Foroud, T.

Understanding the Effects of Prenatal Alcohol Exposure Using Three-Dimensional Facial Imaging
Vol. 34, No. 1, Pages 38–41
Overview: Assessing the Genetic Risk for Alcohol Use Disorders
Vol. 34, No. 3, Pages 266–273

Godin, E.A.

Focus On: Magnetic Resonance–Based Studies of Fetal Alcohol Spectrum Disorders in Animal Models
Vol. 34, No. 1, Pages 99–105

Gossage, J.P.

Maternal Risk Factors for Fetal Alcohol Spectrum Disorders: Not As Simple As It Might Seem
Vol. 34, No. 1, Pages 15–26

Grant, B.F.

Stress and Alcohol: Epidemiologic Evidence
Vol. 34, No. 4, Pages 391–400

Grant, S.G.N.

Discovering Genes Involved in Alcohol Dependence and Other Alcohol Responses: Role of Animal Models
Vol. 34, No. 3, Pages 367–374

Greene, A.M.

Resilience to Meet the Challenge of Addiction: Psychobiology and Clinical Considerations
Vol. 34, No. 4, Pages 506–515

Gruenewald, P.J.

Regulating Availability: How Access to Alcohol Affects Drinking and Problems in Youth and Adults
Vol. 34, No. 2, Pages 248–256

Harris, R.A.

Alcohol Dependence and Genes Encoding α2 and γ1 GABAA Receptor Subunits: Insights From Humans and Mice
Vol. 34, No. 3, Pages 345–354
Using Genetically Engineered Animal Models in the Postgenomic Era to Understand Gene Function in Alcoholism
Vol. 34, No. 3, Pages 282–292

Hasin, D.S.

Stress and Alcohol: Epidemiologic Evidence
Vol. 34, No. 4, Pages 391–400

Hatzenbueller, M.L.

Stress and Alcohol: Epidemiologic Evidence
Vol. 34, No. 4, Pages 391–400

Hawkins, J.D.

Engaging Communities to Prevent Underage Drinking
Vol. 34, No. 2, Pages 167–174

Herman, J.P.

Neural Pathways of Stress Integration: Relevance to Alcohol Abuse
Vol. 34, No. 4, Pages 441–447

Hewitt, B.G.

Fetal Alcohol Spectrum Disorders: Research Challenges and Opportunities
Vol. 34, No. 1, Pages 4–14

Higley, A.E.

Treatment of Alcohol Dependence With Drug Antagonists of the Stress Response
Vol. 34, No. 4, Pages 516–521

Hiller-Sturmhöefel, S.

Translating Family-Focused Prevention Science Into Public Health Impact: Illustrations From Partnership-Based Research
Vol. 34, No. 2, Pages 188–203

Hurley, T.D.

Genes Encoding Enzymes Involved in Ethanol Metabolism
Vol. 34, No. 3, Pages 339–344

Iacoviello, B.M.

Resilience to Meet the Challenge of Addiction: Psychobiology and Clinical Considerations
Vol. 34, No. 4, Pages 506–515

Idrus, N.M.

Fetal Alcohol Spectrum Disorders: Experimental Treatments and Strategies for Intervention
Vol. 34, No. 1, Pages 76–85

Kendler, K.S.

The Impact of Gene–Environment Interaction on Alcohol Use Disorders
Vol. 34, No. 3, Pages 318–324

Keyes, K.M.

Stress and Alcohol: Epidemiologic Evidence
Vol. 34, No. 4, Pages 391–400

Kobor, M.S.

Focus On: Epigenetics and Fetal Alcohol Spectrum Disorder
Vol. 34, No. 1, Pages 29–37

Koob, G.F.

Treatment of Alcohol Dependence With Drug Antagonists of the Stress Response
Vol. 34, No. 4, Pages 516–521

**Figure:**
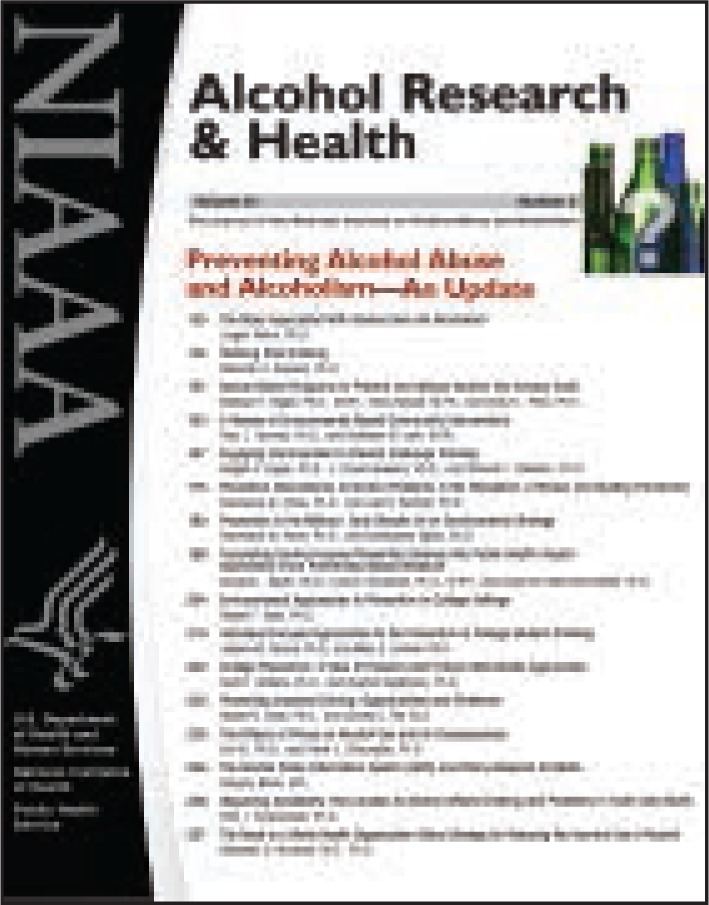
Preventing Alcohol Abuse and Alcoholism—An Update

Kozell, L.B.

Discovering Genes Involved in Alcohol Dependence and Other Alcohol Responses: Role of Animal Models
Vol. 34, No. 3, Pages 367–374

Larimer, M.E.

Individual-Focused Approaches to the Prevention of College Student Drinking
Vol. 34, No. 2, Pages 210–221

Lawson, Q.B.

Resilience to Meet the Challenge of Addiction: Psychobiology and Clinical Considerations
Vol. 34, No. 4, Pages 506–515

Lenk, K.M.

A Review of Environmental-Based Community Interventions
Vol. 34, No. 2, Pages 163–166

Maier, S.E.

Combination Drug Use and Risk for Fetal Harm
Vol. 34, No. 1, Pages 27–28

Mason, B.J.

Treatment of Alcohol Dependence With Drug Antagonists of the Stress Response
Vol. 34, No. 4, Pages 516–521

Mattson, S.N.

The Quest for a Neurobehavioral Profile of Heavy Prenatal Alcohol Exposure
Vol. 34, No. 1, Pages 51–56

May, P.A.

Maternal Risk Factors for Fetal Alcohol Spectrum Disorders: Not As Simple As It Might Seem
Vol. 34, No. 1, Pages 15–26

Miles, M.F.

Identifying Gene Networks Underlying the Neurobiology of Ethanol and Alcoholism
Vol. 34, No. 3, Pages 306–317

Milner, L.C.

Discovering Genes Involved in Alcohol Dependence and Other Alcohol Responses: Role of Animal Models
Vol. 34, No. 3, Pages 367–374

Monteiro, M.G.

The Road to a World Health Organization Global Strategy for Reducing the Harmful Use of Alcohol
Vol. 34, No. 2, Pages 257–260

Moonat, S.

Stress, Epigenetics, and Alcoholism
Vol. 34, No. 4, Pages 495–505

Neighbors, C.

College Prevention: A View of Present (and Future) Web-Based Approaches
Vol. 34, No. 2, Pages 222–224

Neumeister, A.

Resilience to Meet the Challenge of Addiction: Psychobiology and Clinical Considerations
Vol. 34, No. 4, Pages 506–515

Neusel, E.

School-Based Programs to Prevent and Reduce Alcohol Use Among Youth
Vol. 34, No. 2, Pages 157–162

Noronha, A.

Using Genetically Engineered Animal Models in the Postgenomic Era to Understand Gene Function in Alcoholism
Vol. 34, No. 3, Pages 282–292

Nuñez, S.C.

Focus On: Structural and Functional Brain Abnormalities in Fetal Alcohol Spectrum Disorders
Vol. 34, No. 1, Pages 121–131

Nymberg, C.

Genetic and Environmental Determinants of Stress Responding
Vol. 34, No. 4, Pages 484–494

O’Connor, M.J.

Behavioral Interventions for Children and Adolescents With Fetal Alcohol Spectrum Disorders
Vol. 34, No. 1, Pages 64–75

O’Leary-Moore, S.K.

Focus On: Magnetic Resonance–Based Studies of Fetal Alcohol Spectrum Disorders in Animal Models
Vol. 34, No. 1, Pages 99–105

Paley, B.

Behavioral Interventions for Children and Adolescents With Fetal Alcohol Spectrum Disorders
Vol. 34, No. 1, Pages 64–75

Pandey, S.C.

Epigenetics—Beyond the Genome in Alcoholism
Vol. 34, No. 3, Pages 293–305
Stress, Epigenetics, and Alcoholism
Vol. 34, No. 4, Pages 495–505

Parnell, S.E.

Focus On: Magnetic Resonance–Based Studies of Fetal Alcohol Spectrum Disorders in Animal Models
Vol. 34, No. 1, Pages 99–105

Perry, C.L.

School-Based Programs to Prevent and Reduce Alcohol Use Among Youth
Vol. 34, No. 2, Pages 157–162

Phillips, T.J.

Overview: Assessing the Genetic Risk for Alcohol Use Disorders
Vol. 34, No. 3, Pages 266–273

Puglia, M.G.

Focus On: Neurotransmitter Systems
Vol. 34, No. 1, Pages 106–120

Randall, C.L.

Anxiety and Alcohol Use Disorders: Comorbidity and Treatment Considerations
Vol. 34, No. 4, Pages 414–431

Rehm, J.

The Risks Associated With Alcohol Use and Alcoholism
Vol. 34, No. 2, Pages 135–143

Reilly, M.T.

Using Genetically Engineered Animal Models in the Postgenomic Era to Understand Gene Function in Alcoholism
Vol. 34, No. 3, Pages 282–292

Riley, E.P.

The Quest for a Neurobehavioral Profile of Heavy Prenatal Alcohol Exposure
Vol. 34, No. 1, Pages 51–56

Roussotte, F.

Focus On: Structural and Functional Brain Abnormalities in Fetal Alcohol Spectrum Disorders
Vol. 34, No. 1, Pages 121–131

Sakharkar, A.J.

Epigenetics—Beyond the Genome in Alcoholism
Vol. 34, No. 3, Pages 293–305

Saltz, R.F.

Environmental Approaches to Prevention in College Settings
Vol. 34, No. 2, Pages 204–209

Sarkar, D.K.

Circadian Genes, the Stress Axis, and Alcoholism
Vol. 34, No. 3, Pages 362–366

Savage, D.D.

Focus On: Biomarkers of Fetal Alcohol Exposure and Fetal Alcohol Effects
Vol. 34, No. 1, Pages 56–63

Saxena, A.

Resilience to Meet the Challenge of Addiction: Psychobiology and Clinical Considerations
Vol. 34, No. 4, Pages 506–515

Schainker, L.M.

Translating Family-Focused Prevention Science Into Public Health Impact: Illustrations From Partnership-Based Research
Vol. 34, No. 2, Pages 188–203

Schumm, J.A.

Alcohol and Stress in the Military
Vol. 34, No. 4, Pages 401–407

Schumman, G.

Genetic and Environmental Determinants of Stress Responding
Vol. 34, No. 4, Pages 484–494

Sinha, R.

Clinical Laboratory Stressors Used to Study Alcohol–Stress Relationships
Vol. 34, No. 4, Pages 459–467
How Does Stress Lead to Risk of Alcohol Relapse?
Vol. 34, No. 4, Pages 432–440

Smith, J.P.

Anxiety and Alcohol Use Disorders: Comorbidity and Treatment Considerations
Vol. 34, No. 4, Pages 414–431

Sokol, R.J.

Prenatal Alcohol Exposure and Miscarriage, Stillbirth, Preterm Delivery, and Sudden Infant Death Syndrome
Vol. 34, No. 1, Pages 86–91

Sowell, E.R.

Focus On: Structural and Functional Brain Abnormalities in Fetal Alcohol Spectrum Disorders
Vol. 34, No. 1, Pages 121–131

Spera, C.

Prevention in the Military: Early Results of an Environmental Strategy
Vol. 34, No. 2, Pages 180–182

Spoth, R.L.

Translating Family-Focused Prevention Science Into Public Health Impact: Illustrations From Partnership-Based Research
Vol. 34, No. 2, Pages 188–203

Starkman, B.G.

Epigenetics—Beyond the Genome in Alcoholism
Vol. 34, No. 3, Pages 293–305

Stephens, M.A.C.

Stress and the HPA Axis: Role of Glucocorticoids in Alcohol Dependence
Vol. 34, No. 4, Pages 468–483

Stigler, M.H.

School-Based Programs to Prevent and Reduce Alcohol Use Among Youth
Vol. 34, No. 2, Pages 157–162

**Figure:**
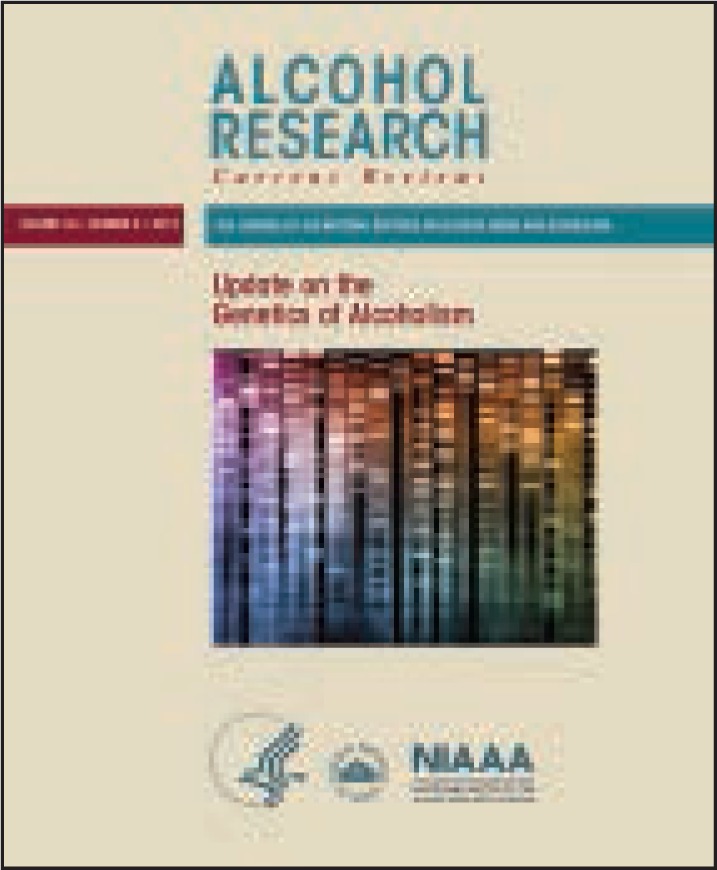
Update on the Genetics of Alcoholism

Sulik, K.K.

Focus On: Magnetic Resonance–Based Studies of Fetal Alcohol Spectrum Disorders in Animal Models
Vol. 34, No. 1, Pages 99–105

Thomas, J.D.

Fetal Alcohol Spectrum Disorders: Experimental Treatments and Strategies for Intervention
Vol. 34, No. 1, Pages 76–85
Fetal Alcohol Spectrum Disorders: Research Challenges and Opportunities
Vol. 34, No. 1, Pages 4–14

Thomas, S.

Clinical Laboratory Stressors Used to Study Alcohol–Stress Relationships
Vol. 34, No. 4, Pages 459–467

Toomey, T.L.

A Review of Environmental-Based Community Interventions
Vol. 34, No. 2, Pages 163–166

Uhart, M.

Clinical Laboratory Stressors Used to Study Alcohol–Stress Relationships
Vol. 34, No. 4, Pages 459–467

Valenzuela, C.F.

Focus On: Neurotransmitter Systems
Vol. 34, No. 1, Pages 106–120

Voas, R.B.

Preventing Impaired Driving: Opportunities and Problems
Vol. 34, No. 2, Pages 225–235

Walters, S.T.

College Prevention: A View of Present (and Future) Web-Based Approaches
Vol. 34, No. 2, Pages 222–224

Wand, G.

Stress and the HPA Axis: Role of Glucocorticoids in Alcohol Dependence
Vol. 34, No. 4, Pages 468–483

Warren, K.J.

Fetal Alcohol Spectrum Disorders: Research Challenges and Opportunities
Vol. 34, No. 1, Pages 4–14

Weinberg, J.

Focus On: Epigenetics and Fetal Alcohol Spectrum Disorder
Vol. 34, No. 1, Pages 29–37

Wetherill, L.

Understanding the Effects of Prenatal Alcohol Exposure Using Three-Dimensional Facial Imaging
Vol. 34, No. 1, Pages 38–41

Wilson, S.E.

Focus On: The Use of Animal Models for the Study of Fetal Alcohol Spectrum Disorders
Vol. 34, No. 1, Pages 92–98

Wolen, A.R.

Identifying Gene Networks Underlying the Neurobiology of Ethanol and Alcoholism
Vol. 34, No. 3, Pages 306–317

Xu, X.

The Effects of Prices on Alcohol Use and Its Consequences
Vol. 34, No. 2, Pages 236–245

Zucca, S.

Focus On: Neurotransmitter Systems Vol. 34, No. 1, Pages 106–120

## Index of Articles

A Review of Environmental-Based Community Interventions

T.L. Toomey and K.M. Lenk
Vol. 34, No. 2, Pages 163–166

Alcohol and Stress in the Military

J.A. Schumm and K.M. Chard
Vol. 34, No. 4, Pages 401–407

Alcohol Dependence and Genes Encoding α2 and γ1 GABAA Receptor Subunits: Insights From Humans and Mice

C.M. Borghese and R.A. Harris
Vol. 34, No. 3, Pages 345–354

Anxiety and Alcohol Use Disorders: Comorbidity and Treatment Considerations

J.P. Smith and C.L. Randall
Vol. 34, No. 4, Pages 414–431

Behavioral Interventions for Children and Adolescents With Fetal Alcohol Spectrum Disorders

B. Paley and M.J. O’Connor
Vol. 34, No. 1, Pages 64–75

Bridging Animal and Human Models: Translating From (and to) Animal Genetics

A.M. Barkley-Levenson and J.C. Crabbe
Vol. 34, No. 3, Pages 325–335

Childhood Trauma, Posttraumatic Stress Disorder, and Alcohol Dependence

K.T. Brady and S.E. Back
Vol. 34, No. 4, Pages 408–413

Circadian Genes, the Stress Axis, and Alcoholism

D.K. Sarkar
Vol. 34, No. 3, Pages 362–366

Clinical Laboratory Stressors Used to Study Alcohol–Stress Relationships

S.S. Thomas, A.K. Bacon, R. Sinha, M. Uhart, and B. Adinoff
Vol. 34, No. 4, Pages 459–467

College Prevention: A View of Present (and Future) Web-Based Approaches

S.T. Walters and C. Neighbors
Vol. 34, No. 2, Pages 222–224

Combination Drug Use and Risk for Fetal Harm

W.-J.A. Chen and S.E. Maier
Vol. 34, No. 1, Pages 27–28

Defining Risk Drinking

D.A. Dawson
Vol. 34, No. 2, Pages 144–156

Discovering Genes Involved in Alcohol Dependence and Other Alcohol Responses: Role of Animal Models

K.J. Buck, L.C. Milner, D.L. Denmark, S.G.N. Grant, and L.B. Kozell
Vol. 34, No. 3, Pages 367–374

Discriminating the Effects of Prenatal Alcohol Exposure From Other Behavioral and Learning Disorders

C.D. Coles
Vol. 34, No. 1, Pages 42–50

Effects of Alcohol Dependence and Withdrawal on Stress Responsiveness and Alcohol Consumption

H.C. Becker
Vol. 34, No. 4, Pages 449–458

Engaging Communities to Prevent Underage Drinking

A.A. Fagan, J.D. Hawkins, and R.F. Catalano
Vol. 34, No. 2, Pages 167–174

Environmental Approaches to Prevention in College Settings

R.F. Saltz
Vol. 34, No. 2, Pages 204–209

Epigenetics—Beyond the Genome in Alcoholism

B.G. Starkman, A.J. Sakharkar, and S.C. Pandey
Vol. 34, No. 3, Pages 293–305

Fetal Alcohol Spectrum Disorders: Experimental Treatments and Strategies for Intervention

N.M. Idrus and J.D. Thomas
Vol. 34, No. 1, Pages 76–85

Fetal Alcohol Spectrum Disorders: Research Challenges and Opportunities

K.R. Warren, B.G. Hewitt, and J.D. Thomas
Vol. 34, No. 1, Pages 4–14

Focus On: Biomarkers of Fetal Alcohol Exposure and Fetal Alcohol Effects

L.N. Bakhireva and D.D. Savage
Vol. 34, No. 1, Pages 56–63

Focus On: Epigenetics and Fetal Alcohol Spectrum Disorder

M.S. Kobor and J. Weinberg
Vol. 34, No. 1, Pages 29–37

Focus On: Magnetic Resonance–Based Studies of Fetal Alcohol Spectrum Disorders in Animal Models

S.K. O’Leary-Moore, S.E. Parnell, E.A. Godin, and K.K. Sulik
Vol. 34, No. 1, Pages 99–105

Focus On: Neurotransmitter Systems

C.F. Valenzuela, M.P. Puglia, and S. Zucca
Vol. 34, No. 1, Pages 106–120

Focus On: Structural and Functional Brain Abnormalities in Fetal Alcohol Spectrum Disorders

S.C. Nuñez, F. Roussotte, and E.R. Sowell
Vol. 34, No. 1, Pages 121–131

Focus On: The Use of Animal Models for the Study of Fetal Alcohol Spectrum Disorders

S.E. Wilson and T.A. Cudd
Vol. 34, No. 1, Pages 92–98

Genetic and Environmental Determinants of Stress Responding

T.-K. Clarke, C. Nymberg, and G. Schumann
Vol. 34, No. 4, Pages 484–494

Genes Contributing to the Development of Alcoholism: An Overview

H.J. Edenberg
Vol. 34, No. 3, Pages 336–338

Genes Encoding Enzymes Involved in Ethanol Metabolism

T.D. Hurley and H.J. Edenberg
Vol. 34, No. 3, Pages 339–344

How Does Stress Lead to Risk of Alcohol Relapse?

R. Sinha
Vol. 34, No. 4, Pages 432–440

Identifying Gene Networks Underlying the Neurobiology of Ethanol and Alcoholism

A.R. Wolen and M.F. Miles
Vol. 34, No. 3, Pages 306–317

Identifying Genetic Variation for Alcohol Dependence

A. Agrawal and L.J. Bierut
Vol. 34, No. 3, Pages 274–281

Immune Function Genes, Genetics, and the Neurobiology of Addiction

F.T. Crews
Vol. 34, No. 3, Pages 355–361

Individual-Focused Approaches to the Prevention of College Student Drinking

J.M. Cronce and M.E. Larimer
Vol. 34, No. 2, Pages 210–221

Maternal Risk Factors for Fetal Alcohol Spectrum Disorders: Not As Simple As It Might Seem

P.A. May and J.P. Gossage
Vol. 34, No. 1, Pages 15–26

Neural Pathways of Stress Integration: Relevance to Alcohol Abuse

J.P. Herman
Vol. 34, No. 4, Pages 441–447

Overview: Assessing the Genetic Risk for Alcohol Use Disorders

T. Foroud and T.J. Phillips
Vol. 34, No. 3, Pages 266–273

Overview: Stress and Alcohol Use Disorders Revisited

R.M. Anthenelli
Vol. 34, No. 4, Pages 386–390

Prenatal Alcohol Exposure and Miscarriage, Stillbirth, Preterm Delivery, and Sudden Infant Death Syndrome

B.A. Bailey and R.J. Sokol
Vol. 34, No. 1, Pages 86–91

Preventing Impaired Driving: Opportunities and Problems

R.B. Voas and J.C. Fell
Vol. 34, No. 2, Pages 225–235

Prevention in the Military: Early Results of an Environmental Strategy

G.M. Ames and C. Spera
Vol. 34, No. 2, Pages 180–182

Prevention Interventions of Alcohol Problems in the Workplace: A Review and Guiding Framework

G.M. Ames and J.B. Bennett
Vol. 34, No. 2, Pages 175–187

Regulating Availability: How Access to Alcohol Affects Drinking and Problems in Youth and Adults

P.J. Gruenewald
Vol. 34, No. 2, Pages 248–256

Resilience to Meet the Challenge of Addiction: Psychobiology and Clinical Considerations

T.N. Alim, W.B. Lawson, A. Feder, B.M.
Iacoviello, S. Saxena, C.R. Bailey, A.M.
Greene, and A. Neumeister
Vol. 34, No. 4, Pages 506–515

School-Based Programs to Prevent and Reduce Alcohol Use Among Youth

M.H. Stigler, E. Neusel, and C.L. Perry
Vol. 34, No. 2, Pages 157–162

Stress and Alcohol: Epidemiologic Evidence

K.M. Keyes, M.L. Hatzenbuehler, B.F. Grant, and D.S. Hasin
Vol. 34, No. 4, Pages 391–400

Stress and the HPA Axis: Role of Glucocorticoids in Alcohol Dependence

M.A.C. Stephens and G. Wand
Vol. 34, No. 4, Pages 468–483

Stress, Epigenetics, and Alcoholism

S. Moonat and S.C. Pandey
Vol. 34, No. 4, Pages 495–505

The Alcohol Policy Information System (APIS) and Policy Research At NIAAA

G. Bloss
Vol. 34, No. 2, Pages 246–247

The Effects of Prices on Alcohol Use and Its Consequences

X. Xu and F.J. Chaloupka
Vol. 34, No. 2, Pages 236–245

The Impact of Gene–Environment Interaction on Alcohol Use Disorders

D.M. Dick and K.S. Kendler
Vol. 34, No. 3, Pages 318–324

The Quest for a Neurobehavioral Profile of Heavy Prenatal Alcohol Exposure

S.N. Mattson and E.P. Riley
Vol. 34, No. 1, Pages 51–56

The Risks Associated With Alcohol Use and Alcoholism

J. Rehm
Vol. 34, No. 2, Pages 135–143

The Road to a World Health Organization Global Strategy for Reducing the Harmful Use of Alcohol

M.G. Monteiro
Vol. 34, No. 2, Pages 257–260

Translating Family-Focused Prevention Science Into Public Health Impact: Illustrations From Partnership-Based Research

R.L. Spoth, L.M. Schainker, and S. Hiller-Sturmhöefel
Vol. 34, No. 2, Pages 188–203

Treatment of Alcohol Dependence With Drug Antagonists of the Stress Response

A.E. Higley, G.F. Koob, and B.J. Mason
Vol. 34, No. 4, Pages 516–521

Understanding the Effects of Prenatal Alcohol Exposure Using Three-Dimensional Facial Imaging

L. Wetherill and T. Foroud
Vol. 34, No. 1, Pages 38–41

Using Genetically Engineered Animal Models in the Postgenomic Era to Understand Gene Function in Alcoholism

M.T. Reilly, R.A. Harris, and A. Noronha
Vol. 34, No. 3, Pages 282–292

**Figure:**
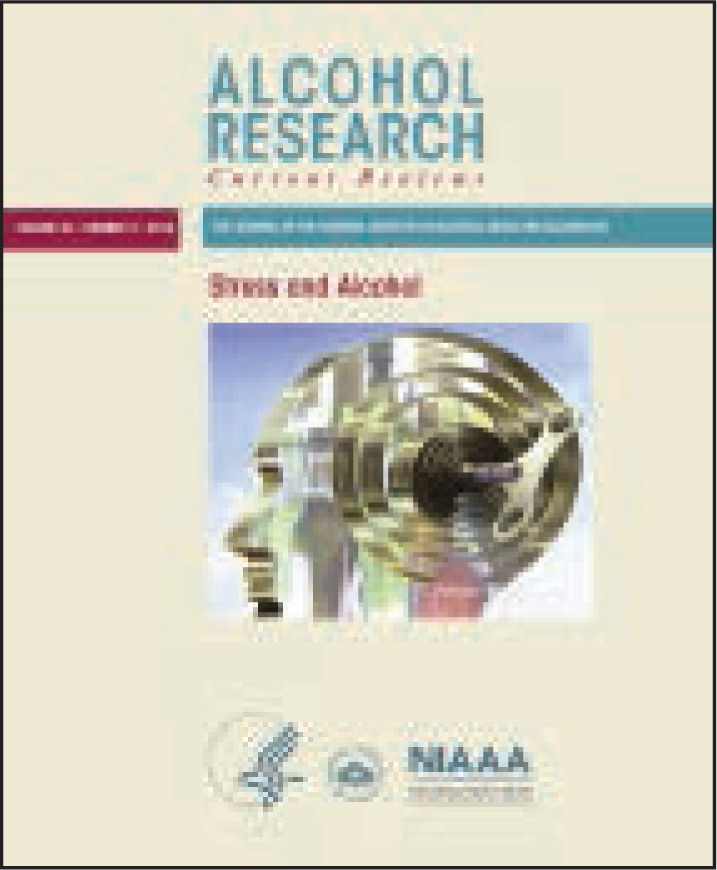
Stress and Alcohol

